# Intraperitoneal Administration of Paclitaxel Combined with S-1 Plus Oxaliplatin as Induction Therapy for Patients with Advanced Gastric Cancer with Peritoneal Metastases

**DOI:** 10.1245/s10434-020-09388-4

**Published:** 2020-12-03

**Authors:** Shin Saito, Hironori Yamaguchi, Hideyuki Ohzawa, Hideyo Miyato, Rihito Kanamaru, Kentaro Kurashina, Yoshinori Hosoya, Alan Kawarai Lefor, Naohiro Sata, Joji Kitayama

**Affiliations:** 1 Department of Gastrointestinal Surgery, Jichi Medical University, Tochigi, Japan; 2 Department of Chemotherapy, Jichi Medical University, Tochigi, Japan

## Abstract

**Background:**

Intraperitoneal (IP) administration of paclitaxel (PTX) has a great pharmacokinetic advantage to control peritoneal lesions and can be combined with various systemic chemotherapies. In this study, we evaluate the efficacy and tolerability of a combination of IP-PTX and systemic S-1/oxaliplatin (SOX) for induction chemotherapy for patients with peritoneal metastases (PM) from gastric cancer (GC).

**Patients and Methods:**

Patients with GC who were diagnosed as macroscopic PM (P1) or positive peritoneal cytology (CY1) by staging laparoscopy between 2016 and 2019 were enrolled. PTX was IP administered at 40 mg/m^2^ on days 1 and 8. Oxaliplatin was IV administered at 100 mg/m^2^ on day 1, and S-1 was administered at 80 mg/m^2^/day for 14 consecutive days, repeated every 21 days. Survival time and toxicities were retrospectively explored.

**Results:**

Forty-four patients received SOX + IP-PTX with a median (range) of 16 (1–48) courses, although oxaliplatin was suspended due to the hematotoxicity or intolerable peripheral neuropathy in many patients. The 1-year overall survival (OS) rate was 79.5% (95% CI 64.4–88.8%) with median survival time of 25.8 months. Gastrectomy was performed in 20 (45%) patients who showed macroscopic shrinkage of PM with a 1-year OS rate of 100% (95% CI 69.5–100%). Grade 2 and 3 histological responses was achieved in four (20%) and one (5%) patients. Grade 3/4 toxicities included neutropenia (11%), leukopenia (39%), and anemia (14%). There were no treatment-related deaths.

**Conclusions:**

Combination chemotherapy using SOX + IP-PTX regimen is highly effective and recommended as induction chemotherapy for patients with PM from GC.

Peritoneal metastases (PM) are the most frequent type of metastases and site of recurrence in patients with advanced gastric cancer (GC).^1^^–^3 At this time, patients with PM are generally treated with systemic chemotherapy, similar to patients with metastases at other sites. Based on phase 3 trials,^4^^,^^5^ fluoropyrimidine plus platinum agents are used as standard regimen in Asia, while docetaxel or anthracyclines are combined in Western countries.^6^^–^8 However, previous studies focused on patients with PM showed that the effect of systemic chemotherapy alone is limited for peritoneal lesions,^9^^–^11 presumably due to the so called peritoneal-plasma barrier which prevents effective drug delivery from the systemic circulation to peritoneal lesions.^12^ Hyperthermic intraperitoneal chemotherapy (HIPEC) combined with cytoreductive surgery (CRS) have been tried for patients with PM of GC mainly in Western countries. However, unlike PM from other primary malignancies, such as ovarian or colorectal cancer, these aggressive treatments have not resulted in significant survival benefits in patients with GC,^13^^–^15 and high morbidity hampered widespread use of this treatment strategy, except at specialized centers.

Paclitaxel (PTX) is an anticancer drug characterized by efficient transition into the peritoneum via intravenous (IV) administration and has been considered a suitable drug to treat PM from GC.^16^ When PTX is intraperitoneally (IP) administered, it is expected to maintain a high concentration in the peritoneal cavity due to its pharmaceutical characteristics of being hydrophobic with a high molecular weight, and elicit notable antitumor activity against peritoneal deposits with less systemic toxicity.^17^ IP-PTX first attracted attention in the treatment of patients with ovarian cancer, another malignancy commonly associated with PM, and its clinical efficacy was verified by several convincing clinical trials.^18^^–^20 Based on these considerations, we developed a protocol combining weekly IP-PTX with S-1 and IV-PTX for patients with GC with PM, which showed 1-year overall survival (OS) rates of 78% in patients with macroscopic (P1) or microscopic (P0CY1) PM^21^ and 77% in patients with P1 disease.^22^ Subsequently, the promising efficacy of this regimen was supported by the results of a phase III trial (Phoenix-GC trial), although the primary endpoint marginally failed to meet the predetermined level of significance.^23^

Over the course of previous clinical studies, we performed conversion gastrectomy for patients who had disappearance or obvious shrinkage of PM after chemotherapy including IP-PTX, which showed excellent outcomes.^24^^,^^25^ However, effects of S-1 and IV-PTX were less effective against the primary tumor as well as extraperitoneal metastases as compared with the activity against peritoneal lesions, which encouraged us to introduce other systemic regimens combined with IP-PTX. In a previous phaseI/II study (clinical trial information: UMIN000012834), we tried to combine IP-PTX with systemic S-1/oxaliplatin (SOX) and determined the recommended dose of IP-PTX to be 40 mg/m^2^.^26^ In the current study, we reevaluated the efficacy and tolerabilility of this regimen as induction therapy for patients with PM from GC.

## Patients and Methods

### Patients and Treatment

Patients diagnosed with advanced GC underwent staging laparoscopy and were enrolled in this study when macroscopic disseminated metastases (P1) or positive peritoneal cytology (CY1) was confirmed. The eligibility criteria included: (1) histologically proven unresectable or recurrent gastric adenocarcinoma, (2) peritoneal dissemination diagnosed by staging laparoscopy or computed tomography (CT) scan, (3) age > 20 years, (4) performance status (Eastern Cooperative Oncology Group) of 0–2, (5) adequate bone marrow function (leukocyte count, 3000–12,000/mm^3^), (6) hemoglobin, > 8.0 g/dL and platelet count, > 100,000/mm^3^, (7) adequate liver function (total serum bilirubin level < 2.0 mg/dL and serum transaminases < 100/UI), (8) adequate renal function (serum creatinine level within the upper limit of the normal), (9) expected survival period of > 3 months, (10) absence of metastases to distant organ sites (liver, lungs, or bone) except the ovary, and (11) no other active concomitant malignancies or other major morbidities. Three patients who had received prior chemotherapy with S1 + oxaliplatin before coming to our institute were included in this study. Written informed consent was obtained from all patients. This study was carried out in accordance with the Declaration of Helsinki of 1975 and was approved by Institutional Review Board of Jichi Medical University.

In those patients, a peritoneal access port was placed in the subcutaneous space of the lower abdomen with a catheter placed in the pelvic cavity as described previously.^27^ The Peritoneal Cancer Index (PCI), which quantitatively combines the distribution of intraperitoneal tumors,^28^ was determined at time of staging laparoscopy. PTX was IP administered through peritoneal access port at 40 mg/m^2^ on days 1 and 8 based on the results of a previous study.^26^ PTX was diluted in 1 L of normal saline, then administered through implanted peritoneal access port over 1 h. Oxaliplatin was IV administered at 100 mg/m^2^ on day 1 and S-1 was administered at 80 mg/m^2^ for 14 consecutive days, followed by 7 days of rest. After several courses of the combination chemotherapy, second look laparoscopy was performed in patients who appeared to have a clinical response. Response was subjectively determined by comparing the number and size of PM with the previous laparoscopic session. When the macroscopic shrinkage of peritoneal lesions was confirmed, together with negative peritoneal cytology in multiple tests and no other distant metastases developed, gastrectomy with lymph node dissection was performed as cytoreductive surgery.

### Assessment of Response and Toxicity

Prior to each course of treatment, medical history, physical examination, laboratory studies (blood cell count, electrolyte levels, liver and renal function tests, and urinalysis), and chest radiography were performed. Gastroendoscopy, upper gastrointestinal radiography, and CT scan were performed to define extent of disease and response. Tumor responses were evaluated after every three courses of treatment and categorized based on the RECIST guidelines (version 1.1). The volume of malignant ascites and peritoneal cytology were also considered to assess anti-tumor effects. In accordance with the Japanese Classification of Gastric Carcinoma,^29^ volume of ascites was assessed by radiologists based on CT scan. On the first day of each treatment course, ascites or peritoneal lavage fluid was collected through a peritoneal access port and cytology was evaluated. Toxicity was graded according to the National Cancer Institute’s Common Terminology Criteria for Adverse Events, version 4.0 Institute.

### Statistical Analysis

The 1-year and 2-year overall survival (OS) rates were estimated according to the Kaplan–Meier method. Overall survival curves were compared using the log rank test, and a *p* value < 0.05 was considered to be statistically significant.

## Results

From January 2016 to March 2019, a total of 44 patients with PM from gastric cancer received SOX + IP-PTX as induction chemotherapy who were fully evaluated for survival and toxicity. Patient characteristics are presented in Table 1. All patients underwent staging laparoscopy and PCI score was evaluated during the initial investigative laparoscopy before IP chemotherapy was administrated.

**Table 1:** Patient characteristics

Characteristic	Total patients (%) (*n* = 44)	Patients who underwent conversion surgey (%) (*n* = 20)
Age (years)	64 (37–77)^a^	68 (38–74)^a^
Gender		
Male	24 (55%)	11 (55%)
Female	20 (45%)	9 (45%)
ECOG performance status		
0	37 (84%)	19 (95%)
1	5 (11%)	1 (5%)
2	1 (2%)	0 (0%)
Previous chemotherapy		
Received	3 (7%)	1 (5%)
Not received	41 (93%)	19 (95%)
Macroscopic type		
Type 4 (diffuse infiltrative)	29 (66%)	11 (55%)
Non-type 4	15 (34%)	9 (45%)
Histological type		
Differentiated	3 (7%)	1 (5%)
Mixed	9 (21%)	4 (20%)
Undifferentiated	32 (73%)	15 (75%)
Extent of peritoneal metastases (JCGC 12th edition)^b^		
P0CY1	2 (5%)	2 (10%)
P_1_	5 (11%)	5 (25%)
P_2_	5 (11%)	2 (10%)
P_3_	32 (73%)	11 (55%)
PCI score	14 (0–39)^a^	6 (0–25)^a^
0–9	19	13
10–20	10	5
21–39	15	2
Peritoneal cytology (CY)		
Positive	29 (66%)	11 (55%)
Negative	15 (34%)	9 (45%)
Other distant metastasis		
Ovary	2 (5%)	0 (0%)
Para-aortic lymph nodes	1 (2%)	0 (0%)
Absent	41 (93%)	20 (100%)

^a^Shown as media (range)

^b^*CY1* peritoneal cytology findings positive for carcinoma cells

*ECOG* Eastern Cooperative Oncology Group, *JCGC* Japanese classification of gastric carcinoma, *P0* no peritoneal metastasis, *P*_*1*_ metastases immediately adjacent to the stomach, *P*_*2*_ several scattered metastases within the peritoneal cavity, *P*_*3*_ numerous metastases throughout the peritoneal cavity, *PCI* peritoneal cancer index

### Outcomes and Response

The median length of follow-up for censored cases was 27.1 months (12.8–50.6 months). A median of 16 courses (range 1–48) of combination chemotherapy were given. In many cases, however, IV oxaliplatin was suspended due to hematotoxicity or intolerable peripheral neuropathy, and the full regimen was given for a median of 6 courses (1–18).

**Fig. 1:**
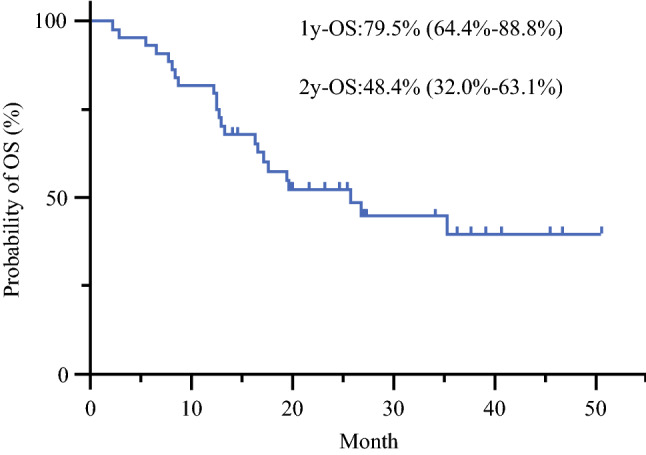
Kaplan–Meier analysis of overall survival (OS) of all patients (*n* = 44)

**Fig. 2:**
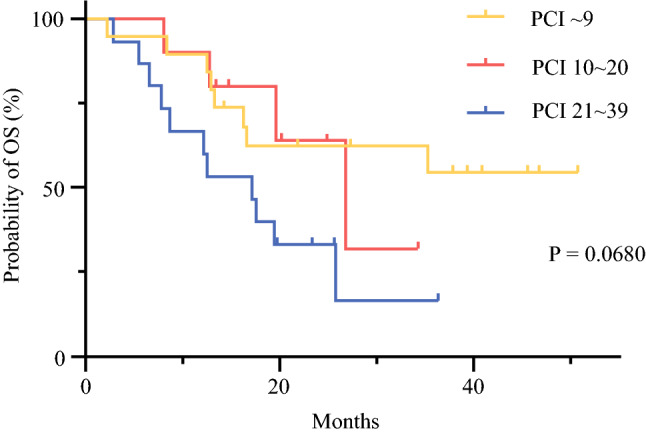
Kaplan–Meier analysis of overall survival (OS) of patients with peritoneal cancer index (PCI) scores of 1–9 (*n *= 19), 10–20 (*n* = 10), and 20–39 (*n* = 15)

**Fig. 3:**
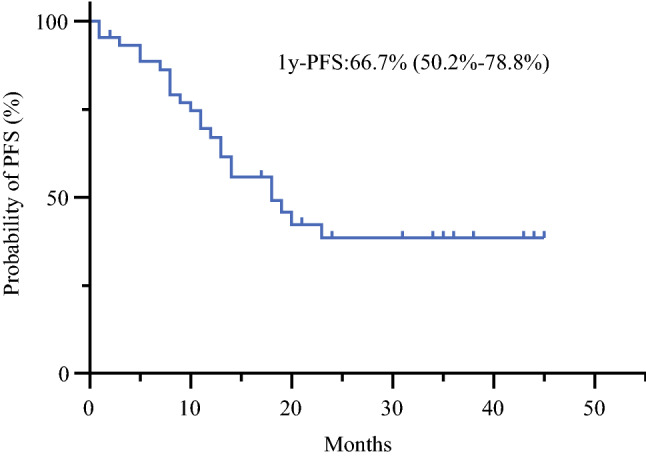
Kaplan–Meier analysis of progression free overall survival (PFS) of patients of all patients (*n* = 44)

Figure 1 shows OS time after introduction of combination chemotherapy for all 44 patients enrolled in this study. The 1-year OS rate was 79.5% (95% CI 64.4–88.8%), and the 2-year OS rate was 48.4% (95% CI 32.0–63.1%). MST was 25.8 months (95% CI 16.3 months to not reached). As shown in Fig. 2, OS in patients with low PCI tended to be better, and half of patients with PCI < 10 lived more than 3 years. However, MSTs of patients with PCI scores of 10–20 and > 20 were 28 and 18 months, respectively, and the difference was not statistically different (*p* = 0.0680). Only three patients had measurable disease according to RECIST criteria, but two of these (67%) showed objective responses. Malignant ascites disappeared or decreased in 15/22 (68%) patients. Peritoneal cytology became negative in 25/29 (86%) patients. During the course of the combination chemotherapy, 4 patients developed distant metastasis at extra-peritoneal organs and 19 patients showed the progression in peritoneal lesions, determined by the appearance of clinical symptoms such as intestinal or urinary obstruction and ascites. The 1-year progression free survival (PFS) rate was 18.2 months with 1-year PFS of 66.7% (95% CI 50.2–78.8%) and median PFS of 18.2 months (Fig.3).

Gastrectomy (conversion surgery) was performed in 20/44 (45%) patients. The patient profiles and surgical results are shown in Tables 1 and 2, respectively. A median of 9 (2–16) courses of chemotherapy were given before surgery but again oxaliplatin was suspended in 13/20 (65%) patients, and the full regimen was given for a median of 4 (2–11) courses. Combined resections of small intestine or invaded liver were performed in two patients, while neither extended peritonectomy nor intraoperative intraperitoneal chemotherapy with or without hyperthermia was performed. A D2 lymph node dissection was performed in one, and D1 dissection in 19 patients which omitted the prophylactic dissection of splenic hilar lymph nodes. Postoperative leakage (Clavien–Dindo grade II) occurred in one patient who was subsequently treated nonoperatively. There were no treatment-related deaths.

**Table 2:** Results of conversion surgery

Variables	Number of patients (%)
Operative procedure	
Total gastrectomy	18 (90%)
Distal gastrectomy	2 (10%)
Combined resection	
Small intestine	1 (5%)
Liver	1 (5%)
Gastrectomy only	18 (90%)
Lymph node dissection	
D1	19 (95%)
D2	1 (5%)
Postoperative complications	
Anastomotic leakage	1 (5%)
Residual tumor status	
R0 (no residual tumor)	14 (70%)
R1 (microscopic residual tumor)	4 (20%)
R2 (macroscopic residual tumor)	2 (10%)
Histological response^a^	
Grade 1a	8 (40%)
Grade 1b	7 (35%)
Grade 2	4 (20%)
Grade 3	1 (5%)

^a^The histological response of the primary tumor was classified according to the Japanese classification of gastric carcinoma 14th edition and 3rd English edition. Grade 1a viable tumor cells occupy ≥ 2/3 of tumorous area, Grade 1b viable tumor cells occupy ≥ 1/3 of tumorous area, Grade 2 viable tumor cells occupy < 1/3 of tumorous area, Grade 3 no viable tumor

**Fig. 4:**
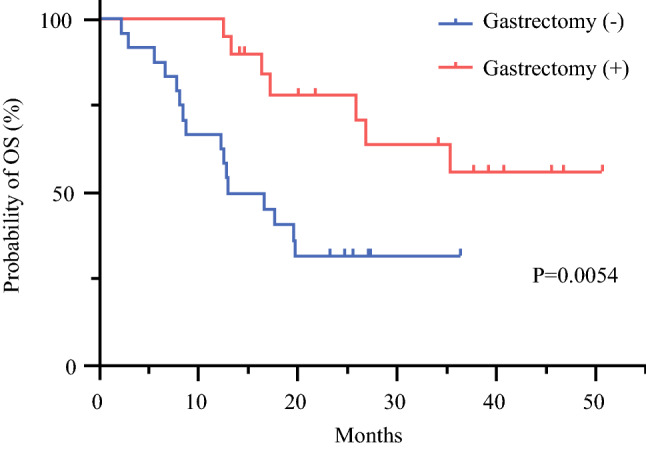
Kaplan–Meier plot analysis of overall survival (OS) of patients who did (*n* = 20) and did not (*n* = 24) undergo conversion gastrectomy

After resection, 14/20 patients (70%) had no residual tumor (R0), while microscopic tumor remained in the resection stump or biopsied scar-like areas on the peritoneal surface in 4/20 patients (20%) (R1), and macroscopic metastatic nodules could not be completely removed during surgery in 2 patients (10%) (R2). According to the histological examination, grade 2 or grade 3 responses in resected primary tumors were observed in 4/20 (20%) and 1/20 (5%) patients, respectively. As shown in Fig. 4, their 1-year and 2-year OS rates were 100% (95% CI 69.5–100%) and 70.9% (95% CI 42.9–87.0%) without reaching MST. The 1-year OS rate of the patients who did not undergo conversion gastrectomy was 62.5% (95% CI 42.9–87.0%) with a MST of 12.9 months (95% CI 8.4–19.7 months), which was significantly lower than those in patients who underwent gastrectomy (*p* < 0.01).

### Safety

Adverse events are presented in Table 3. Frequently occurring grade 3/4 toxic effects included neutropenia (11%), leukopenia (39%), and anemia (14%). Peripheral sensory neuropathy was observed in 24 patients (55%), but the frequency of serious adverse events was low due to using a reduced dose or withholding of systemic oxaliplatin. Oxaliplatin was suspended after a median of 6 courses (range 1–18). Complications related to the peritoneal access device included obstruction of the intraperitoneal catheter in three patients (7%) and infection of the access port in three patients (7%), who needed surgical intervention. No patients developed abdominal pain, or any other toxicity related to IP infusion. No chemotherapy-related mortality was observed.

**Table 3:** Adverse events among all-treated patient set

Grade (CTCAE v 4.0)	1, 2	3	4	% of grade 3/4
Leukopenia	17	3	2	11
Neutropenia	10	13	4	39
Anemia	31	3	3	14
Thrombocytopenia	11	1		2
Fatigue	12	1		2
Anorexia	8			
Nausea	7			
Vomiting	2	1		2
Diarrhea	4	1		2
Rash	1			
Mucositis	2			
Febrile neutropenia		2		5
Peripheral sensory neuropathy	23	1		2
Port-related complication				
Infection		3		7
Obstruction		3		7

*CTCAE* Common Terminology Criteria for Adverse Events

## Discussion

Repeated IP administration of PTX using an implantable peritoneal access port is a reasonable strategy to control peritoneal lesions from a pharmacokinetic perspective and the combination of IP-PTX with S-1 and intravenous (IV) PTX is a promising treatment protocol for treatment of patients of PM from GC.^21^^–^24 However, this regimen is less effective for treatment of extraperitoneal lesions than for PM. The S1/oxaliplatin (SOX) regimen has significant efficacy with acceptable toxicity and is now considered as one of the standard regimens for the first-line treatment of patients with metastatic GC in Asia.^30^^–^32 In particular, this regimen has strong antitumour activity (pCR rate 33%, pRR 83%, pathological downstaging 67%),^33^ which might be more suitable for induction chemotherapy for conversion surgery. A new regimen combining IP-PTX with SOX was developed in order to maximize the systemic effects as well as the local effects against PM.^26^

In this study, we used this regimen as the induction chemotherapy for 44 patients with PM from GC and evaluated the safety of this regimen. The most common toxicities of this regimen were neutropenia, leukopenia, and peripheral sensory neuropathy. The frequency of neurotoxicity was high, but most patients tolerated it if administration of oxaliplatin was modified by occasional skip or dose reduction. Indeed, in most patients, oxaliplatin was withdrawn and S-1 plus IP-PTX was administrated during a later course of combined chemotherapy. This suggests that the recommended dose of IP-PTX (40 mg/m^2^) determined in the previous study should perhaps be reduced in this protocol. However, the 1-year and 2-year OS rates of all patients were 79.5% and 48.4%, respectively. Outcomes tended to be better in patients with low PCI score. Outcomes as well as the profile of adverse events are almost identical to those in previous studies with IP-PTX with S-1/PTX.^21^^–^23 From these observations, it is suggested that IP-PTX can be combined with various systemic chemotherapeutic drugs.

In cases which showed marked shrinkage of PM in second look laparoscopy, gastrectomy was performed in 45% of patients at 6–48 weeks (median 27 weeks) after initiation of combination chemotherapy. Accordingly, R0 resection was performed in 70% patients, however, complete histological response (grade 3) was achieved in only one (5%) patient. Response rates were lower than expected and not so different from that of the previous series using IP-PTX plus S-1/PTX regimen.^25^ This might be related to the fact that the total dose of systemic oxaliplatin administrated was reduced from the original plan in many patients. However, outcomes of pateints who underwent gastrectomy were excellent with a 1-year OS rate of 100%, and MST was not reached, which is much better than that in patients who did not undergo gastrectomy (1-year OS = 62.5%, MST = 12.9). Although this result suggests a possibility that gastrectomy may contribute to prolongation of survival, it is not clear evidence due to selection bias. A comparative study is necessary to clarify clinical significance of conversion gastrectomy.

The strategy of using neoadjuvant intraperitoneal and systemic chemotherapy (NIPS) followed by gastrectomy has been used for treatment of patients with PM of GC for many years. Yonemura et al. reported that the 1-year OS rate and MST were 67.4% and 15.0 months, respectively, in patients who were treated with NIPS with S-1 and IP administration of docetaxel (DTX) and cisplatin followed by cytoreductive surgery.^34^ Fujiwara et al. also reported a 1-year OS rate and MST of 76% and 24.6 months, respectively, in patients who underwent gastrectomy after S-1 + IP-DTX.^35^ The 1-year OS and MST of the patients who undergo gastrectomy after treatment with IP-PTX plus S-1/PTX were 73.3% and 30.5 months, respectively.^25^ Outcomes of patients in this study exceed those results. In the present series, many patients could continue IP-PTX receiving a median of 14 (1–48) courses even after surgery, although systemic oxaliplatin was often suspended during the treatment cycle, which may lead to favorable outcomes. In fact, two patients who underwent R2 resection received IP-PTX for 10 and 24 courses and survived for 14 and 26 months, respectively. This suggests that the repetition of IP-PTX is important for prolonging survival in these patients.

In the IP-PTX and S1/PTX combination chemotherapy regimen, we have treated many patients for whom PM had been controlled for years while the primary tumor or other distant metastases progressed within months, which hampered the continuation of chemotherapy. Therefore, gastrectomy in these patients might have been effective to prevent clinical symptoms caused by regrowth of primary tumor or extraperitoneal lesions and may contribute to maintaining quality of life during the entire treatment course. In that sense, gastrectomy in this study is not definitive “conversion surgery” resulting in curability, but might be more appropriately referred as “interval debulking surgery” as in the treatment of ovarian cancer.^36^

In conclusion, IP-PTX combined with S1/oxaliplatin, although dose reduction is necessary in some cases, can be used for induction chemotherapy for treatment of patients with PM from GC. Gastrectomy performed after an excellent response in PM may contribute to the improvement of patient outcomes. The indication criteria to perform resection are the most crucial issue in the future. In the present study, we performed gastrectomy only in patients who showed obvious shrinkage of PM by laparoscopic examination as well as having negative peritoneal cytology. Judging from the results of this study, the strategy employed appears to be reasonable. However, laparoscopy under general anesthesia induces surgical stress which might have adverse effects from an oncological perspective. Further studies to verify the indication, as well as to determine the appropriate timing of surgery, are warranted. Discovery of biomarkers to define the patient population in whom surgery can result in real survival benefit would be ideal.
